# A downscaling and bias correction method for climate model ensemble simulations of local-scale hourly precipitation

**DOI:** 10.1038/s41598-023-36489-3

**Published:** 2023-06-09

**Authors:** Takao Yoshikane, Kei Yoshimura

**Affiliations:** Institute of Industrial Science, The University of Tokyo, 5-1-5, Kashiwanoha, Kashiwa-Shi, Chiba 277-8574 Japan

**Keywords:** Attribution, Hydrology

## Abstract

Ensemble simulations of climate models are used to assess the impact of climate change on precipitation, and require downscaling at the local scale. Statistical downscaling methods have been used to estimate daily and monthly precipitation from observed and simulated data. Downscaling of short-term precipitation data is necessary for more accurate prediction of extreme precipitation events and related disasters at the regional level. In this study, we developed and investigated the performance of a downscaling method for climate model simulations of hourly precipitation. Our method was designed to recognize time-varying precipitation systems that can be represented at the same resolution as the numerical model. Downscaling improved the estimation of the spatial distribution of hourly precipitation frequency, monthly average, and 99th percentile values. The climate change in precipitation amount and frequency were shown in almost all areas by using the 50 ensemble averages of estimated precipitation, although the natural variability was too large to compare with observations. The changes in precipitation were consistent with simulations. Therefore, our downscaling method improved the evaluation of the climatic characteristics of extreme precipitation events and more comprehensively represented the influence of local factors, such as topography, which have been difficult to evaluate using previous methods.

## Introduction

Detailed predictions of regional precipitation are necessary to accurately estimate the risk of water-related disasters and availability of fresh-water water resources under climate change^1^. Because of a lack of resolution in climate models, dynamic and statistical downscaling methods are used to estimate changes in local weather using climate model outputs^2^. Dynamic downscaling applies the output results of a global climate model to a high-resolution numerical model, which requires substantial computing power. Statistical methods are based on observed linear regressions between precipitation and a range of atmospheric variables^1,2^. Statistical methods are often used to estimate daily or monthly precipitation from observed and simulated data rather than to estimate hourly precipitation based on climate model simulations, unlike the dynamic downscaling method. In general, hourly precipitation is associated with mesoscale precipitation systems and formed by interactions between local factors, such as topography and time-varying atmospheric fields (e.g. orographic rainfall)^3,4^. However, statistical methods are usually incapable of recognizing detailed temporal patterns in precipitation systems and ill-suited for estimating the spatial distributions of hourly precipitation frequencies^5,6^. Without a correct estimate, it is difficult to accurately assess the impact of climate change on regional precipitation patterns^5^.

Downscaling methods using machine learning have recently been developed^7–19^. These methods can accommodate more complex explanatory variables and estimate precipitation with higher levels of accuracy. However, most of these methods do not allow for the estimation of hourly precipitation. Typically, it is difficult to estimate hourly precipitation because even a small difference in atmospheric fields can change the distribution of precipitation due to the nonlinearity of the precipitation process. Therefore, a method that recognizes subtle differences in weather patterns is needed to estimate hourly precipitation. This could be achieved with machine learning methods using forecast model outputs and observational data, which can potentially be applied to climate models. However, it is unclear whether the patterns recognized by forecast models are applicable to climate models due to the differences in resolution and parameters between these models. Therefore, it is necessary to identify phenomena that are common to both prediction and climate models and applicable to machine learning-based downscaling methods.

In general, numerical models can reproduce meteorological phenomena at five to eight times the grid spacing^20,21^. For example, a model with a grid spacing of ≤ 20 km can potentially reproduce phenomena over 100 km^2^. Numerical models used for weather forecasting can reproduce precipitation systems associated with low-pressure systems, such as warm and cold fronts, as well as the temporal changes in spatially averaged precipitation^22,23^. On the other hand, it is impossible to avoid estimation bias associated with, for example, low data resolution^1,5^. This bias could be reduced based on the pattern of the relationship between the simulated and observed precipitation system^6^.

In this study, we developed a machine learning-based downscaling method that can reduce model bias by recognizing time-varying precipitation systems. Our method is based on the assumption that a model with an equivalent or higher resolution can reproduce a precipitation system with the same characteristics. Therefore, the patterns of the relationship between the observed and simulated precipitation distribution produced by a weather forecast model could be applied to other models, such as climate models. We investigated the performance of the method by applying the recognized patterns to climate model products from the Database for Policy Decision-Making for Future Climate Change (d4PDF)^24,25^. The d4PDF project was undertaken to elucidate the impact on climate change characteristics, which is based on a large number of ensemble runs that reduces the influence of natural variability. In this project, historical climate simulations with global climate models confirmed an upward trend in temperature from 1950 to 2011, similar to observations. Downscaling using a 20-km resolution regional model was conducted to investigate precipitation characteristics around Japan in detail. However, compared to observations, it is difficult to reproduce precipitation characteristics corresponding to the topography.

**Figure 1:**
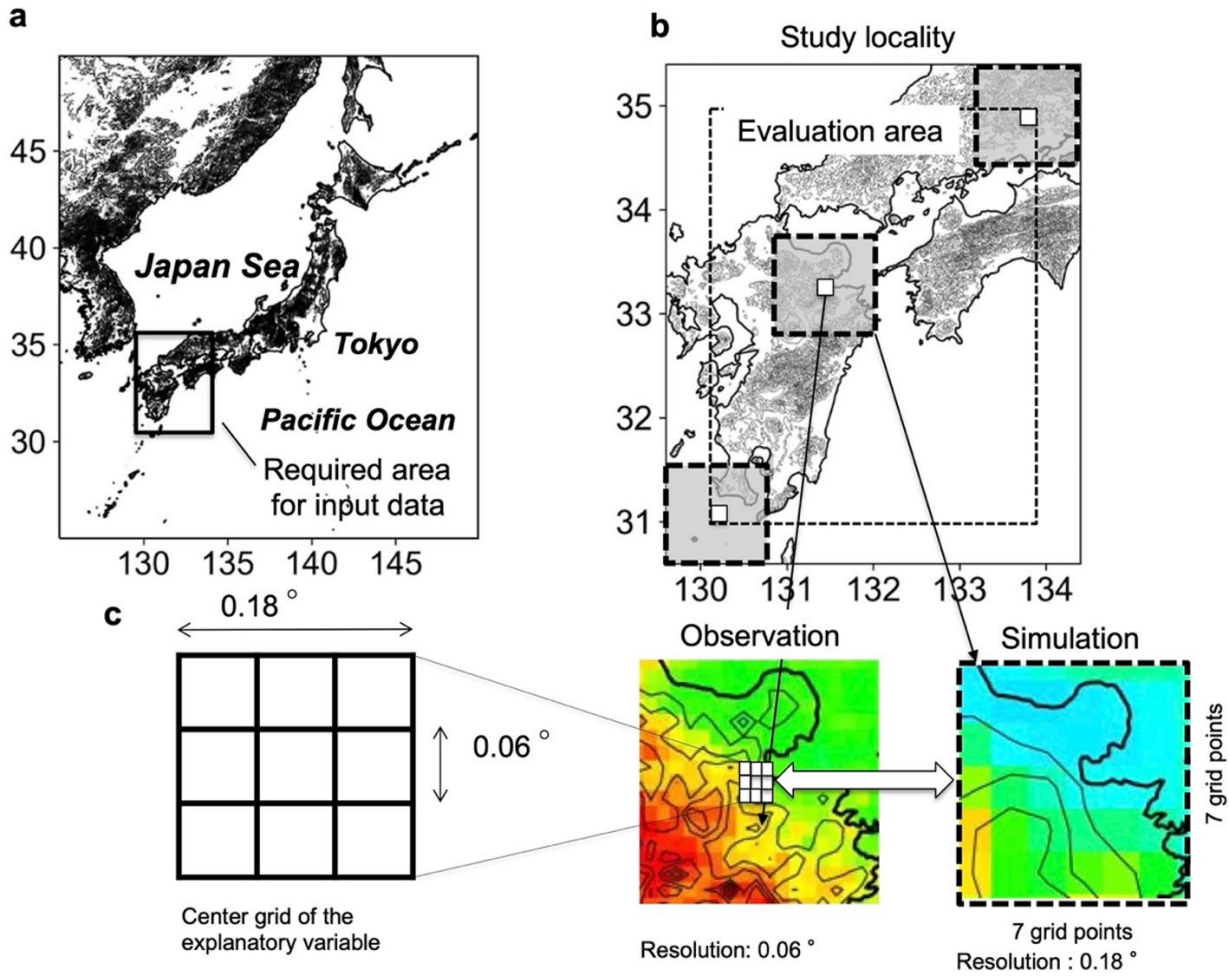
Study locality and downscaling bias correction. (**a**) Required area for input data. (**b**) Evaluation area (dashed line) and the areas of the explanatory variable (shaded area with bold dashed line: 7 × 7 grid points of simulation). (**c**) Grid structure for downscaling from 0.18° to 0.06°. The grid point with 0.18°-squared area (centre grid of explanatory variable) is divided into nine grid points (3 × 3 grids) with a 0.06°-squared area (corresponding to observed precipitation resolution). Precipitation at the downscaled grid points was estimated by the common explanatory variable. The maps were created using python3-matplotlib (version 3.7.1, https://matplotlib.org/) and cartopy (version 0.21.1, https://scitools.org.uk/cartopy). Topographic data of U.S. Geological Survey (USGS) (http://www.usgs.gov) and Japan Meteorological Agency (JMA) were used. Made with Natural Earth. Free vector and raster map data @ naturalearthdata.com. (http://www.naturalearthdata.com/about/terms-of-use/).

Our approach is ultimately aimed at accurately assessing the impact of climate change on local precipitation, though it is also expected to promote the prediction of water-related disasters and fresh-water availability. In this study, we applied the machine learning downscaling method to estimate local precipitation in southwestern Japan (Fig. 1), which recently experienced many water-related disasters due to severe precipitation events^26–28^. Accordingly, we used data of 20 km around Japan downscale from a 60 km (“Methods” and Fig. S1) and confirmed the application of the downscaling model in climate models. The workflow of our method is shown in Fig. S2.

## Validity of downscaling method using machine learning

To assess whether the assumption of the machine learning-based downscaling method is met, we applied the precipitation distribution patterns predicted by forecast model 1 (FM1, 5 km grid spacing) to forecast model 2 (FM2, 20 km grid spacing) and a climate model (CM, d4PDF) (“Methods”). FM1 and FM2 are forecast models developed by the Japan Meteorological Agency (JMA), which provide forecast data including analytical values as initial values using a 5 km resolution nonhydrostatic model and a 20 km resolution global spectral model, respectively. The original data of FM1 were upscaled to 0.18° to match the resolution of FM2 and the CM. Observed precipitation data, upscaled to 0.06°, were used as the objective variable for computational efficiency (Fig. 1). First, the machine learning method was used to correct for bias in the spatial distribution of simulated hourly precipitation. We estimated precipitation in the target year by training the observed precipitation and FM1 data in a 7 × 7 grid point area centred on the grid point including observed precipitation as explanatory variables, excluding year. Second, a quantile mapping method was applied to the machine learning estimates to address underestimation and provide quantitative corrections. Cumulative distribution functions were created using the machine learning estimates and observations except for the target year, and the CDF-transform method was used to estimate precipitation in the target year (MLQM-FM1). To investigate the performance of classifiers created with the upscaled FM1 data, the estimation was repeated using FM2 data (MLQM-FM2). Finally, the estimates obtained by applying hourly precipitation data from the climate model to the discriminator created in FM1 were applied to the CDF created with the machine learning estimates from FM1 and observations (MLQM-CM) (Methods, Figs. S1 and S2).

We focused on the long-term spatial distribution of hourly frequency, monthly average, and 99th percentile values (corresponding to rainfall intensity) of hourly precipitation. We only considered precipitation events ≥ 1 mm h^−1^. The spatial distributions of hourly precipitation frequency was used to verify the performance (bias correction) of the machine learning-based downscaling method. The 99th percentile value of hourly precipitation was used to evaluate changes in the intensity of extreme precipitation events. The amount of precipitation is affected by both the frequency and intensity of precipitation events^29^.

We determined the temporal variations in the area-averaged hourly precipitation using MLQM-FM1, MLQM-FM2, and radar observations (OBS) in the study locality for July of 2008 to 2018 (Fig. S3 and “Methods”). The temporal variations in MLQM-FM1 and MLQM-FM2 were well estimated compared with the OBS, while the estimates varied slightly between models. The distributions of hourly precipitation frequency, monthly precipitation, and 99th percentile values in MLQM-FM1 and MLQM-FM2 were comparable to the observed distributions (Fig. S4). Therefore, the downscaling method reduced estimation bias. Figure S5 shows the relationship among the spatial distributions of hourly precipitation frequency, monthly precipitation, and 99th percentile values of hourly precipitation between the observations and the values estimated using the machine learning methods (MLQM-FM1 and MLQM-FM2) or the simulations in FM1 and FM2. Although there was a slight tendency to underestimate, the accuracy of estimated precipitation (MLQM-FM1 and MLQM-FM2) was greatly improved by the downscaling method, suggesting that the method can be applied to the other forecast models by upscaling the precipitation product to be equivalent to the resolution of the model.

## Downscaling of precipitation simulated by a climate model

**Figure 2:**
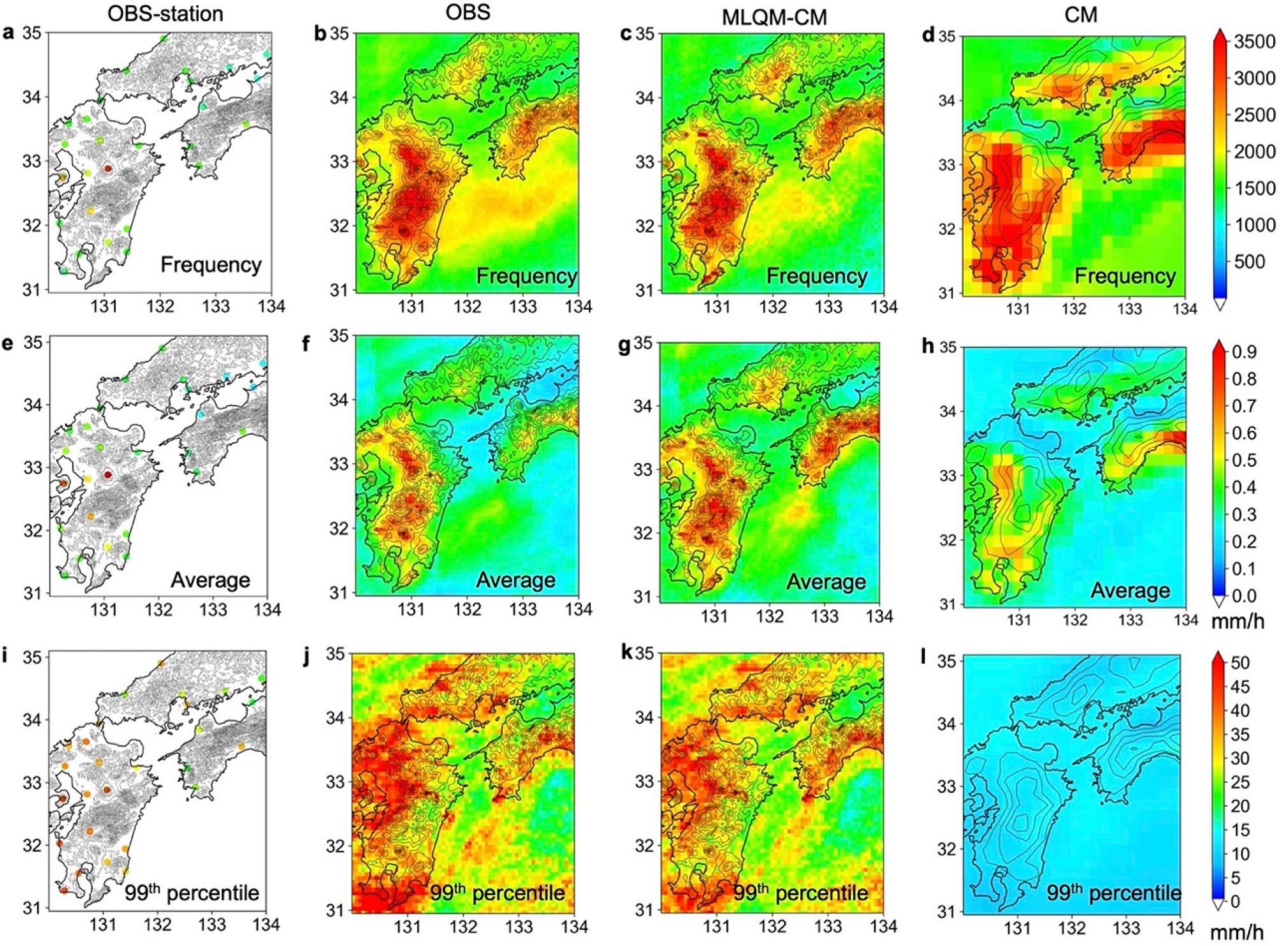
Spatial distribution of precipitation. (**a**) Observations from meteorological stations (OBS-station); (**b**) radar observations (OBS); (**c**) values estimated by machine learning-based downscaling method (MLQM-CM); (**d**) values simulated by d4PDF with a 20 km grid (CM); (**e**–**h**) monthly precipitation; and (**i**–**l**) 99th percentile values of hourly precipitation. All values, except for those of OBS, are associated with the period from 1982 to 2011; OBS data are from 2007 to 2018, and the frequency was adjusted by extending the term 2.5 times. The maps were created using python3-matplotlib (version 3.7.1, https://matplotlib.org/) and cartopy (version 0.21.1, https://scitools.org.uk/cartopy). Topographic data of U.S. Geological Survey (USGS) (http://www.usgs.gov) and Japan Meteorological Agency (JMA) were used. Made with Natural Earth. Free vector and raster map data @ naturalearthdata.com. (http://www.naturalearthdata.com/about/terms-of-use/).

**Figure 3:**
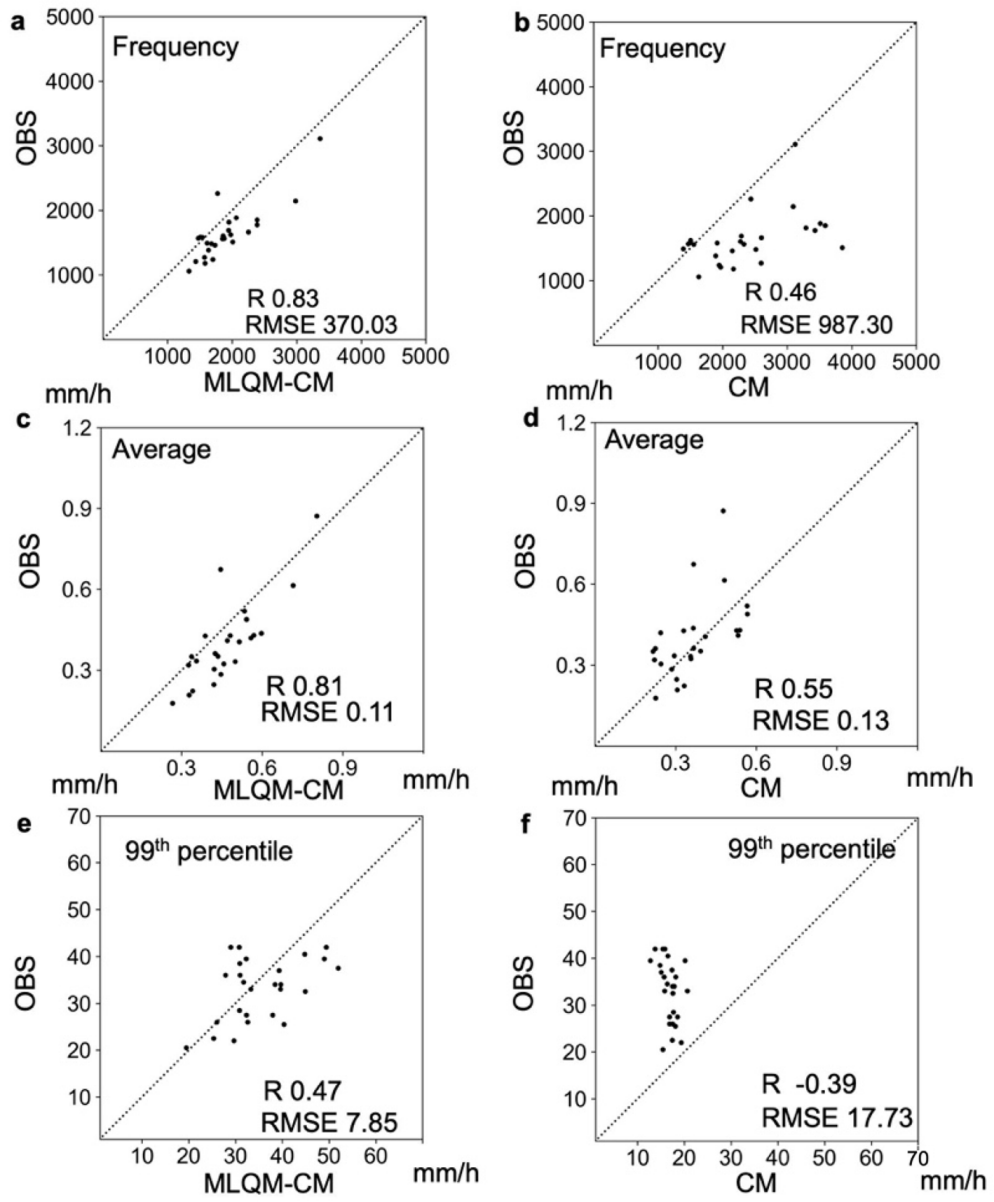
Validation of estimated precipitation using weather station data over 30 years. The relationship of the frequency between observations at 26 stations over 30 years and (**a**) MLQM-CM and (**b**) CM; (**c**, **d**) the relationship associated with monthly precipitation; and (**e**, **f**) the relationship of the 99th percentile values of hourly precipitation from 1982 to 2011.

Figure 2 shows the spatial distributions of hourly precipitation frequencies, monthly precipitation, and 99th percentile values of hourly precipitation of the observations, estimations using the machine learning method (MLQM-CM), and climate model (CM). In the simulations, the frequency of precipitation tended to be higher on the plains and the lower mountainous areas (Fig. 2d), while it tended to be higher in the higher mountainous areas. On the other hand, the frequency distribution estimated by the machine learning method was comparable to that for the observations (Fig. 2c). The monthly precipitation distributions showed similar characteristics to those of the spatial distribution of hourly precipitation frequency, while the amount of precipitation in the simulations was underestimated overall compared to those in machine learning estimates (Fig. 2f,h). The distributions of 99th percentile values of hourly precipitation were significantly underestimated by the CM (Fig. 2i), while the spatial distribution of precipitation was well estimated by the machine learning-based downscaling method (Fig. 2k). Figure S6 shows the Q–Q plots for the hourly precipitation frequency, monthly precipitation, and 99th percentile values of hourly precipitation between the OBS and MLQM-CM or CM. The correlation coefficients and root mean square errors (RMSEs) in the MLQM-CM were larger and smaller than those in the CM, respectively. This result clearly confirmed that the simulated model output varied greatly from the observations, while precipitation estimated by our method corresponded well to that in the observations. Figure 3 shows the Q–Q plots for observations at 26 stations (OBS-station) over 30 years (1982 to 2011). The estimated precipitation was selected at the nearest grid point from each station. The correlation coefficients and RMSEs of the MLQM-CM were larger and smaller than those in the CM, respectively. The correlation coefficients between the OBS and precipitation estimated by the MLQM-CM for all ensemble experiments (30-year dataset) was > 0.65, indicating that the long-term distribution of precipitation was well estimated. Compared to the OBS-station, the 99th percentile values showed a decrease in correlation coefficient, but the monthly precipitation and frequencies showed high correlation coefficients (Fig. S7).

**Figure 4:**
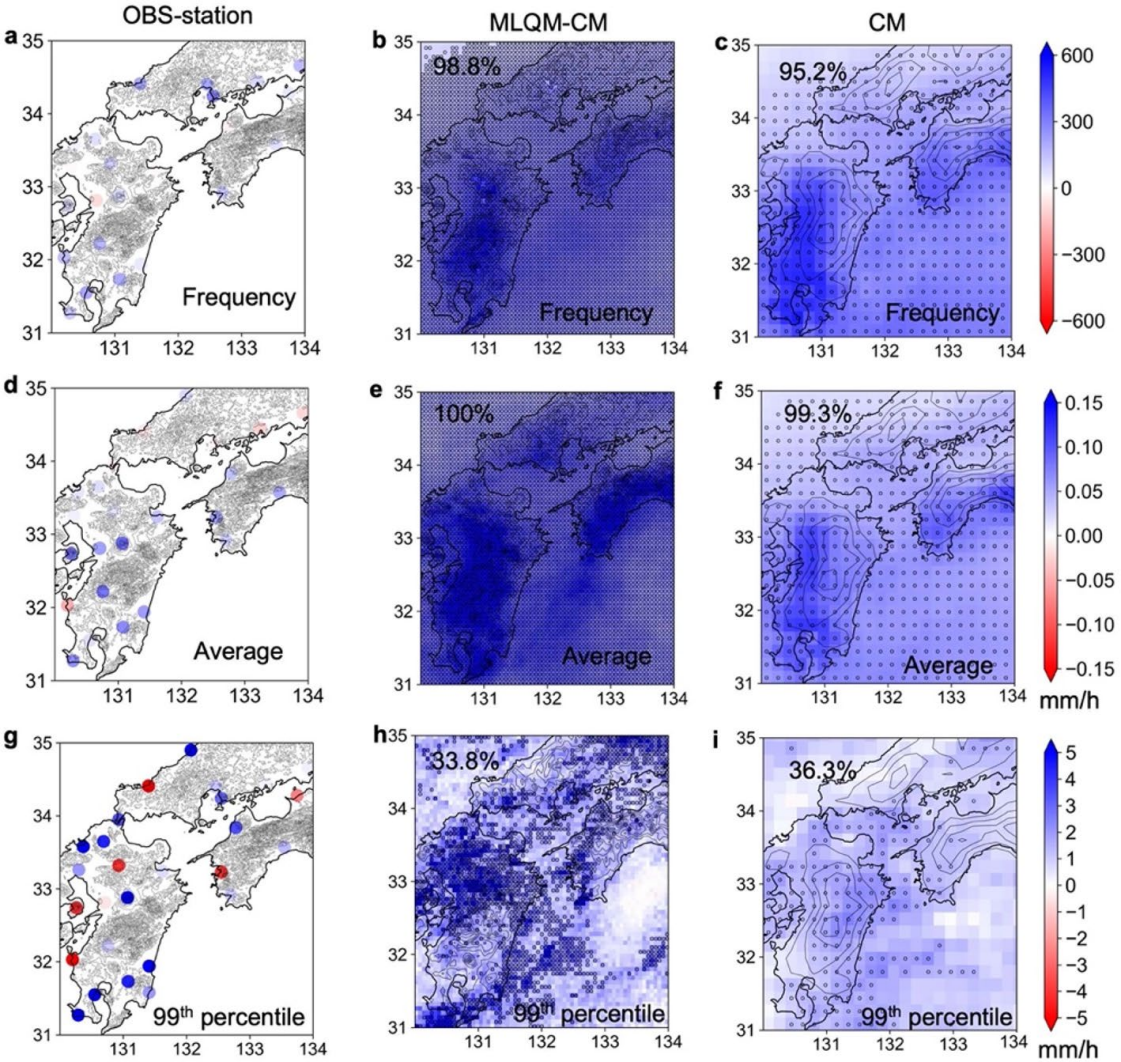
Effects if climate change on precipitation. Differences in the spatial distributions of 99th percentile, monthly average, and frequency of precipitation between observations and 50 ensemble runs in the MLQM-CM and CM for the period 1952 to 1981 and 1982 to 2011. (**a**, **d**, **g**) Observed precipitation at 26 sites (OBS-station), (**b**, **e**, **h**) MLQM-CM, and (**c**, **f**, **i**) CM. The circle markers show statistically significant differences of 50 ensemble average values with 95 confidence intervals (Welch’s *t*-test). The percentage shows the ratio of significantly different grids from the total. The maps were created using python3-matplotlib (version 3.7.1, https://matplotlib.org/) and cartopy (version 0.21.1, https://scitools.org.uk/cartopy). Topographic data of U.S. Geological Survey (USGS) (http://www.usgs.gov) and Japan Meteorological Agency (JMA) were used. Made with Natural Earth. Free vector and raster map data @ naturalearthdata.com. (http://www.naturalearthdata.com/about/terms-of-use/).

The 60 years of d4PDF historical climate simulation data were divided into the first 30 years (1952–1981) and the second 30 years (1982 – 2011), and changes in precipitation of climate values were estimated for each of the first and second 30 years. As shown in the d4PDF simulations, temperatures clearly tend to increase in the latter 30 years compared to the first 30 years, so we examined the impact of the temperature increase in the latter 30 years on precipitation. Figure 4 shows the effect of climate change on precipitation based on the differences in the spatial distributions of the 99th percentile, monthly average, and hourly frequency of precipitation between observations and 50 ensemble runs in MLQM-CM and CM from 1952 to 1981 and 1982 to 2011. Under climate change, the frequency and monthly average of precipitation increased significantly in almost all areas, and in about 30% of the areas for the 99th percentile values. However, the effect of climate change on OBS-station precipitation patterns was unclear. The variation in the increment ratios of the observations roughly corresponded to the standard deviation of 50 ensembles of the MLQM-CM and CM. Although the quantitative characteristics of the MLQM-CM were somewhat different from those of the CM, the magnitude of the standard deviations was comparable (Fig. S8).

The representative spatial scales of the precipitation systems estimated by our method were estimated by the spatial autocorrelations of hourly precipitation. Fig. S9 shows the total number of grid points with autocorrelations > 0.7, which indicates strong correlations. The spatial scales in the OBS corresponded well to those in FM1, which were ~ 30 km^2^; the scales in FM2 and the CM corresponded to ~ 100 km^2^. The spatial scales in the MLQM-FM1, MLQM-FM2, and MLQM-CM were slightly larger than those in FM2 and the CM.

The characteristics of the precipitation system varied somewhat between high-resolution data (FM1) and low-resolution (FM2) data. To investigate the impact of the upscaled data on precipitation estimation, we repeated the analysis using FM2 data (with approximately the same resolution as the CM) from 1982 to 2011 in ensemble number 1 (Fig. S10, Table S1, and “Methods”). Figure S11 shows the frequency, monthly average, and 99th percentile values of precipitation for MLQM-CM-FM1-ENS1 and MLQM-CM-FM2-ENS1. The spatial distribution of the hourly frequency, monthly average, and 99th percentile values in MLQM-CM-FM2-ENS1 corresponded well to those in MLQM-CM-FM1-ENS1 with high correlation coefficients (Fig. S12), although some quantitative differences were observed.

## Discussion

Compared to the spatial distribution of precipitation frequency and monthly average, the distribution of 99th percentile values varied somewhat. We observed many locations where precipitation frequencies and monthly averages were low, but the 99th percentile was high. In general, the leeward side of mountains is more likely to form downward flow, which suppresses precipitation, while precipitation is promoted on the windward side of the mountain. Considering that wind direction changes and the suppression of precipitation is more pronounced in the foothills on leeward mountain sides, it is reasonable that the frequency of precipitation is smaller in mountainous areas. On the other hand, heavy rainfall is assumed to be determined by characteristics of local topography, regardless of the frequency of rainfall. The strong correlation of 99th percentile values between the MLQM-CM and OBS suggests that this method estimates the suppression or enhancement of precipitation at each location based on the spatial distribution pattern of precipitation. However, the correlation of 99th percentile values between the MLQM-CM and the OBS-station was relatively small. The regional characteristics were so pronounced that the 5 km resolution was insufficient, and the number of heavy rainfall events was small, which may explain this error. However, there is no particularly large discrepancy between OBS and OBS-station (Figs. S6e and 3e), which may indicate that the number of sites was too few to clearly analyse the association with topography (weather stations were mostly located at the foothills of mountains, resulting in a bias).

The spatial distribution of precipitation was comparable with observations for hourly frequency, monthly mean, and 99th percentile values, which indicates that our downscaling method estimated the temporal variation (intensity) of local precipitation corresponding to the precipitation system reproduced by the climate model. The quantile mapping method is based on the application of cumulative distribution functions produced by the machine learning estimates (using a forecast model) and observations for the period from 2008 to 2018. Therefore, if the temporal change (intensity) of precipitation is not properly estimated by the machine learning method, there will be large errors in the long-term precipitation characteristics. The method is designed to recognize patterns in precipitation by considering its basic components (spatial distribution of hourly precipitation). Therefore, the results also showed that the climate model effectively reproduced the climatic characteristics of the frequency and magnitude of precipitation systems.

The difference in the spatial scales of autocorrelation between the models indicated that the 20 km grid models, such as FM2 and CM, were less effective in reproducing smaller scale precipitation systems found in the observations and FM1. In early summer, back-building squall lines often form in the target area, causing localized heavy rainfall^30,31^. Squall lines are formed by individual cumulus convection interacting with ambient atmospheric fields^30–32^. Therefore, the correct prediction of individual cumulus convection would allow for the accurate reproduction of squall lines. In contrast, larger-scale precipitation systems in the range of 3000 to 20,000 km^2^, which are representative of the spatial scales used in our method (MLQM-FM1, MLQM-FM2, and MLQM-CM), are formed under the influence of large-scale atmospheric fields, such as monsoons, tropical cyclones, extratropical cyclones, or quasi-stationary fronts. The spatial scales of precipitation systems are equivalent to meso-β- and meso-α-scale disturbances^33,34^ in association with large-scale disturbances, which are often formed in early summer. In other words, our method more clearly recognizes the patterns of larger precipitation systems associated with large-scale disturbances than those with strong self-generating processes, such as squall lines. The representative autocorrelation scale of ML-FM1 suggests that while FM1 effectively reproduces the characteristics of small-scale precipitation systems, the position, timing, and intensity of precipitation systems do not always correspond to the observations due to the intrinsic nonlinearity of the system. In the quantile mapping method, local hourly precipitation was quantitatively corrected corresponding to the temporal variation of the estimated precipitation. Considering that the squall line is formed by a larger precipitation system, the estimated precipitation characteristics are deemed reasonable.

Climate models cannot reproduce the spatial distribution of precipitation frequency. In general, precipitation characteristics differ between the windward and leeward sides of mountains (orographic precipitation). With the quantile mapping method alone, the hourly precipitation frequency corresponded to the climate model simulations, which can lead to large errors in the precipitation amount and frequency^6^.

The MLQM-CM effectively estimated the long-term characteristics of the spatial distribution of hourly precipitation frequency, monthly average, and 99th percentile values (Figs. 2 and S6). In addition, we did not observe any extreme under- or overestimation with respect to the observed precipitation, and the amount of estimated precipitation was comparable to that in the observations. This suggests that the frequency and intensity of the precipitation systems, which were reproduced by the CM at a 20 km resolution, were the primary drivers of local precipitation in July. In other words, our method can recognize the patterns of precipitation systems in 140 km^2^ and estimate the temporal variability of local precipitation with high accuracy. This also shows that the climate model effectively reproduced the precipitation systems. The method does not correct for biases in the large-scale atmospheric circulation field, such as the storm track of extratropical cyclones, associated with climate models. Therefore, the applicability of the method strongly depends on the ability of the climate model to reproduce large-scale disturbances that are strongly associated with atmospheric circulation fields and local precipitation.

The climate change characteristics of the estimated precipitation (MLQM-CM) were consistent with the simulation (CM), although the spatial distribution of climate change effects differed significantly. On the other hand, the natural variability was too large for comparison with the last 60 years of observations (Fig. S8). The 50 ensemble averages of the estimates and simulations showed significant increases in frequency and monthly averages almost everywhere, but the 99th percentile values increased only at about 30% of all areas (Fig. 4). The increase in extreme precipitation in global climate models is explained by the Clausius-Clapeyron (CC) relationship (7% increase per degree of warming)^35,36^. However, recent studies have also indicated that there is super-Clusius–Clapeyron scaling (above the CC relationship) and sub-Clusius–Clapeyron scaling (below the CC relationship) for extreme precipitation increases^37–39^. As for the sub-Clausius–Clapeyron scaling, it may be related to the water vapor condensation mechanism in the mountains^37^. However, a detailed investigation will be needed to take into account the complex thermodynamic effects in mountains.

In the QM method, precipitation frequency is strongly dependent on simulated values. If there is no precipitation in the simulation, no precipitation correction is made in QM even if there is precipitation in the observation. Therefore, if precipitation spatial distribution characteristics differ significantly due to differences between the model and real terrain, the correction may not be appropriate^6,40^. Our method applies the QM method after correcting the spatial distribution of precipitation by machine learning. Therefore, it can estimate the characteristics of precipitation without the problem of the QM method described above.

In principle, machine learning methods cannot be applied to the estimation of hourly precipitation, which has never been observed. In addition, the rarer the phenomenon, the smaller the sample size and larger the estimation error, which may make accurate evaluation difficult. In this study, the evaluation was made for the 99th percentile value, but the limits of applicability may vary greatly depending on conditions. When making an evaluation, it is necessary to investigate the limits of applicability through comparisons with observed and simulated data.

The climatic characteristics of orographic precipitation are strongly dependent on the frequency and intensity of atmospheric disturbances, which are dominated by large-scale atmospheric circulation patterns^41^. The results of this study indicate that our method can be used to effectively estimate the long-term characteristics of local hourly precipitation. Furthermore, we expect that this downscaling method would allow the prediction of water-related disasters, such as floods, by taking advantage of the ensemble simulations of climate models (d4PDF), which can reproduce a variety of unprecedented extreme event patterns involving multiple precipitation systems or prolonged stagnation of large precipitation zones or tropical cyclones^42,43^.

This method, like dynamic downscaling, is strongly influenced by the atmospheric fields reproduced by the climate model. Therefore, it is expected that the estimations will be affected by differences in physical processes between climate models, such as cumulus convection schemes. Therefore, when applying this method, it is necessary to select a climate model with a small synoptic-scale atmospheric field bias.

## Conclusion

In this study, we developed a machine learning-based downscaling method to estimate regional hourly precipitation linked to climate model simulations by identifying time-varying precipitation systems represented in numerical models with a 20 km resolution. The spatial distribution of hourly frequency, monthly average, and intensity (99th percentile values) of precipitation were well estimated by our method using the outputs of a climate model. We also found that the climatic characteristics of the estimated values corresponded to the simulated results over the entire region. Overall, we found that (1) the climate model could reproduce the climatic characteristics of the observed precipitation system and (2) downscaling and bias correction of temporally variable precipitation could reflect local conditions, such as topography. This suggests that the climatic characteristics of local precipitation strongly depend on the formation pattern of the precipitation system, which changes over the short-term. Accordingly, our method can be applied to climate model downscaling and can estimate the spatial distribution of local precipitation from the outputs of climate models with coarse spatial and temporal resolution while accounting for model limitations. In the future, we plan to apply our method to future projections and climate reconstructions using climate models to study the effects of local climate change and elucidate its mechanisms.

## Methods

### Bias correction and downscaling using machine learning

We used a support vector machine regression model (SVM–SVR)^44^, which was constructed as previously described^6^. SVM is a supervised learning method that uses a subset of the dataset to obtain predictions from support vectors. SVM tries to obtain optimal results by finding the maximal margin hyperplane, which is determined by maximizing the distance between the support vectors. Compared to other ML methods, such as neural networks and random forests, SVM holds many advantages^45–48^. For example, SVR has been shown to perform well even with a small sample size^45^. SVM has been adopted in various fields, such as meteorology, hydrology, disaster management, and water resource management, among others, and has proven to be useful for recognizing rare precipitation events^7,49,50^. The support vector machine library in the scikit-learn system (Epsilon-Support Vector Regression) in scikit-learn 0.24.2 system^51^. In the SVR method, we set the hyperparameters gamma, C, and epsilon; gamma specifies the width of the Gaussian radial basis function (RBF) kernel, whereas C is the penalizing constraint error and epsilon is the width of the insensitive zone^52^. Determination of these hyperparameters is very important to improve the generality of precipitation estimation. The hyperparameters could be configured at each point in the downscaling method; however, determining the optimal parameters requires considerable computational resources^46,53^. To obtain the optimal hyperparameters more effectively, we applied the specified hyperparameter values to all grid cells in the domain according to the following procedure: First, we estimated the optimal hyperparameter values by random search^54^ on some grid points in the domain. The optimal values of gamma, C, and epsilon were found to be approximately 5 × 10^–6^, 10, and 0.001, respectively. We assumed that the same parameters were applicable to all grid cells because they did not vary extensively among the grid points. Next, we investigated the performance of the downscaling method in estimating precipitation based on the correlation coefficients of 49 grid cells, and the coefficients were averaged over every 10 grids. First, the optimal gamma value was estimated using temporary values of C (10) and epsilon (0.001). Second, the optimal C value was obtained using the optimal gamma value and a temporary epsilon value. Third, the optimal epsilon value was obtained using both the optimal gamma and C values. Finally, the optimal gamma was obtained using both the optimal C and epsilon values. The parameters were considered to be optimal if they corresponded to the first estimates or if the correlation coefficients did not clearly improve. The optimal values of gamma, C, and epsilon were approximately 5 × 10^–6^, 10, and 0.001, respectively. Thus, we configured all grid cells using the optimal hyperparameter values.

Observed precipitation upscaled to 0.06° and precipitation output from the Japan Meteorological Agency (JMA) mesoscale numerical model (MSM-GPV) upscaled to 0.18° were used as input data. In this study, the numerical models of MSM-GPV^22^, GSM-GPV^55^, and d4PDF_RCM^24,25^ with different spatial resolutions were used to analyse the input data; the resolution of the explanatory variables was maintained at 0.18°. MSM-GPV and GSM-GPV were used as training and validation data for the machine learning models as FM1 and FM2, respectively. d4PDF_RCM was used for bias correction and downscaling of the climate model simulations. The spatial resolution of the observation data was ~ 1 km, but was upscaled to 0.06° for computational efficiency. We used hourly precipitation for July of 2007 to 2018 and conducted training using all years except for the test year (Fig. S1); for example, to estimate precipitation for the year 2007, data from 2008 to 2018 were used for training. For training, we used the accumulated precipitation from the analysis time to the first hour of every 3 h (0, 3, 6, 9, 12, 15, 18, and 21 UTC) in consideration of the correspondence with observed precipitation. For inference, we used simulated hourly precipitation data for July. We assessed the performance of the explanatory variables in estimating precipitation to find the optimal grid size. Consequently, the precipitation distribution in a region of 7 × 7 grid points (almost a 140 × 140 km^2^ area) was used as the explanatory variable (feature vector), the grid points located at the centre of the region were divided into 3 × 3 grid points, and the corresponding observed precipitation was assigned as the objective variable. The machine learning-based downscaling method was applied to all the observed grid points in the target area (Fig. 1). The hyperparameters were standardised across the entire target area after confirming that the value estimated by a random search did not change within the target area. Moreover, quantitative bias corrections were conducted on the machine learning estimates by applying the quantile mapping method. Another JMA forecast model output (GSM-GPV) with a 20 km grid was used to verify the precipitation patterns estimated by our method.

### d4PDF data

The Database for Policy Decision-Making for Future Climate Change (d4PDF) is a project assessing the impact of climate change^24,25^. In this study, we used d4PDF_RCM, which is a dynamically downscaled version of the global climate model d4PDF_GCM (60 km resolution) with a 20 km resolution in the Japan region. In the historical climate simulations in d4PDF, the sea-surface temperature, sea ice concentration, and sea ice thickness are prescribed as the lower boundary conditions, and the global mean concentrations of greenhouse gases and three-dimensional distributions of ozone and aerosols are prescribed as external forcings. The calculations start from different initial values, and small perturbations are added to the sea ice and SST.

### Machine learning-based downscaling method for the climate model

The recognized pattern of precipitation distributions simulated by the weather forecast model (MSM-GPV)^22^ and observed precipitation (Radar-AMeDAS)^56^ were applied to the hourly precipitation of d4PDF to perform bias correction and downscaling of precipitation. We used hourly precipitation data from 50 ensemble experiments of d4PDF_RCM for July of 1982 to 2011 (Fig. S1). The resolution of the simulated precipitation was adjusted to 0.18° to be consistent with the recognized pattern of the weather forecast model. Precipitation was estimated with a resolution of 0.06° by downscaling to a fine grid (3 × 3) using the same hyperparameters and feature ranges (7 × 7 grid points) used in the weather forecast model. The CDF-transform quantile mapping method^57^ was applied using observed and estimated precipitation from the machine learning-based downscaling method for 2008 to 2018 and estimated precipitation of d4PDF using the recognized pattern.

### Quantile mapping method

In machine learning, accuracy is highly dependent on sample size, which complicates the estimation of heavy rainfall events. Methods such as under-oversampling can be used to correct unbalanced sampling^58^. In this study, a quantile mapping method (CDFt package in R)^59^ was used instead of an under- or oversampling method because of the complexity of the adjustment. The ‘CDFt’ method assumes that a transformation T exists that allows us to translate the CDF of a GCM variable (such as temperature, precipitation, or wind intensity) into the CDF representing the local-scale long-term variable at a given weather station. *F*_*Oh*_ corresponds to the CDF of the observed data at the meteorological station during the past calibration period, and *F*_*Gh*_ is the CDF that bilinearly interpolates the GCM output at the station during the same period. *F*_*Of*_ and *F*_*Gf*_ are CDFs equivalent to *F*_*Oh*_ and *F*_*Gh*_, respectively, but for future (or simply different) periods. Assuming that *F*_*Gf*_ is known (which can be modelled in the future GCM output), the transformation T can be calculated as:

1 $$T(F_{Gh} (x)) = F_{Oh} (x)$$

2 $$T(F_{Gf} (x)) = F_{Of} (x)$$

To model T, we replace *x* in *Gh* in Eq. (1) with *F(u)*, where u is [0, 1]. We then obtain:

3 $$T(u) = F_{Oh} \circ F^{ - 1}_{Gh} (u)$$

Hence, assuming that the relationship (4) will remain valid, the CDF is provided by:

4 $$F_{Of} (x) = T(F_{Gf} (x)) = F_{Oh} \circ F^{ - 1}_{Gh} (u) \circ F_{Gf} (x)$$

In the CDFt package, Eq. (1) reconstructs *F*_*Of*_ from *F*_*Oh*_, *F*_*Gh*_, and *F*_*Gf*_, and Eq. (2) performs quantile mapping from *F*_*Of*_ and *F*_*Gf*_ to correct G_f_. In practice, *F*_*Oh*_, *F*_*Gh*_, and F_Gf_ are estimated using the empirical cumulative distribution function. However, the CDFt method is considered to work correctly only when the observed values of *Oh* and *Gh* have a similar range. In this study, we used the observed and simulated precipitation values from 2008 to 2018 instead of *Oh* and *Gh*. Then, instead of *Gf*, the corrected precipitation values of d4PDF were estimated by applying the d4PDF-org data from 1952 to 2011.

### Spatial autocorrelation

The averaged spatial scales of the precipitation systems estimated by our downscaling method were estimated using the following equation for spatial autocorrelation^60^:

5 $$r_{k,l} = \frac{{\sum\nolimits_{i = 1}^{n} {\left( {x_{k} (i) - \overline{x}_{k} } \right)\left( {x_{l} (i) - \overline{x}_{l} } \right)} }}{{\sqrt {\sum\nolimits_{i = 1}^{n} {\left( {x_{k} (i) - \overline{x}_{k} } \right)^{2} } } \sqrt {\sum\nolimits_{i = 1}^{n} {\left( {x_{l} (i) - \overline{x}_{l} } \right)^{2} } } }}$$

where *r* is the spatial autocorrelation between points *k* and *l*, *n* is the total number of hourly precipitation data points, and *x* is hourly precipitation data. We used the precipitation data for July of 2008 to 2018 for OBS, FM1, MLQM-FM1, FM2, and MLQM-FM2, while the 30-year dataset from 1982 to 2011 was used for an ensemble experiment of the CM and MLQM-CM.

### Validation of the machine learning-based downscaling method using upscaled data as explanatory variables

To evaluate the impact of using upscaled data as explanatory variables on model performance, machine learning-based precipitation estimation was performed using GSM-GPV data at the same resolution as that for the d4PDF data. Unlike the method used for the machine learning-based downscaling for the climate model, precipitation estimation was only performed for the 1982 to 2011 dataset of ensemble number 1. Details of each experiment are shown in Table S1. The experiments were conducted using 11 years of data from 2008 to 2018, since the Japan Meteorological Agency only started to provide GSM-GPV data from 2008.

## Supplementary Information

Supplementary Information.

## Data Availability

The datasets of “Database for Policy Decision-Making for Future Climate Change” (d4PDF) are available from the Data Integration and Analysis System (DIAS) (https://diasjp.net/service/d4pdf-data-download/). The datasets of Radar-AMeDAS, MSM-GPV, and GSM-GPV are available from the Japan Meteorological Business Support Center (JMBSC) (http://www.jmbsc.or.jp/en/index-e.html). The datasets of surface data point (SDP) of JMA are available from the JMA web site (https://www.jma.go.jp/jma/indexe.html). The other datasets used and/or analysed during the current study are available from the corresponding author on reasonable request.
