# Supplementary material for: A downscaling and bias correction method for climate model ensemble simulations of local-scale hourly precipitation
Source: Sci Rep. 2023 Jun 9;13:9412. doi: 10.1038/s41598-023-36489-3 (PMC10256754; doi:10.1038/s41598-023-36489-3)
Supplement: Supplementary file 1 — Supplementary Information. [file 41598_2023_36489_MOESM1_ESM.pdf]

Supplementary information

A downscaling and bias correction method for climate model ensemble simulations of local-scale hourly precipitation

Takao Yoshikane[1]*, Kei Yoshimura[1]

[1]Institute of Industrial Science, The University of Tokyo, 5-1-5, Kashiwanoha, Kashiwa-shi, Chiba, 277-8574, Japan

*Corresponding author. Email takao-y@iis.u-tokyo.ac.jp

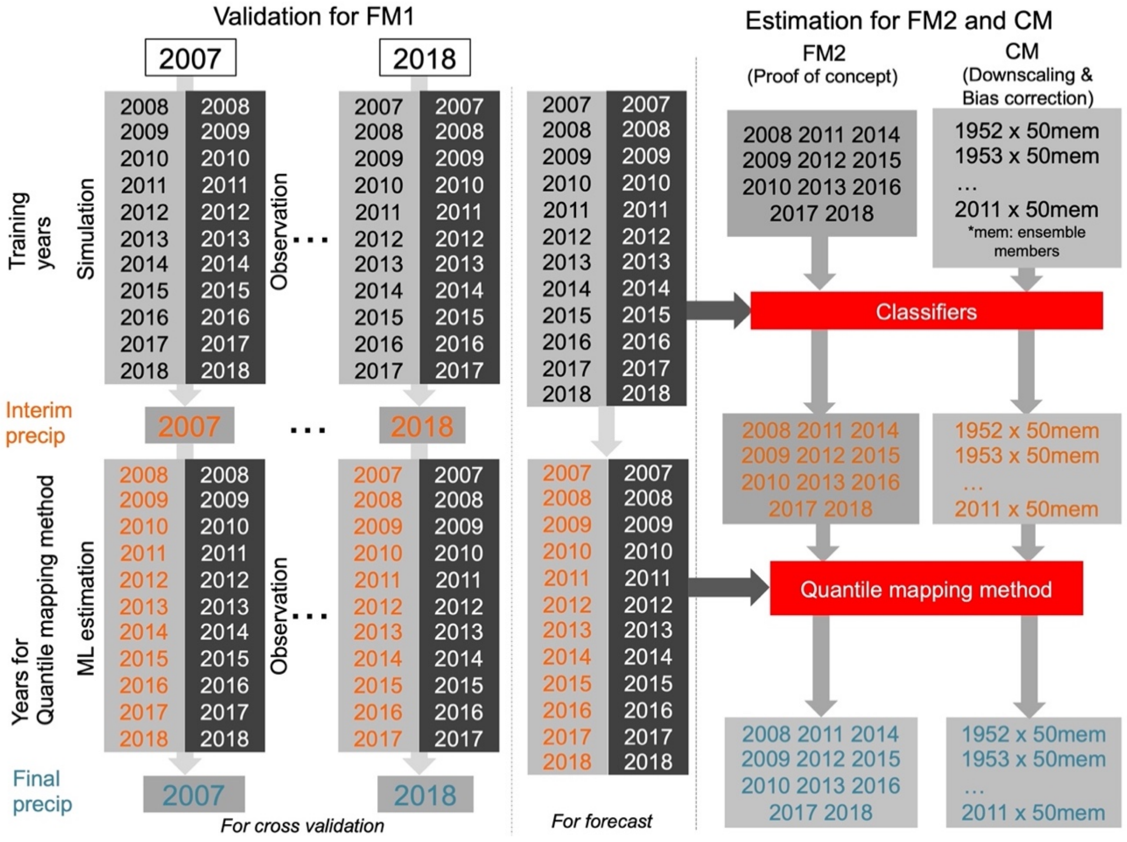

Fig. S1: **Procedure for estimating precipitation using the machine learning-based downscaling method.** In the validation of the weather forecast models, the training terms of the simulations (forecast model) and observations (radar data) are from 2007 to 2018, excluding the estimation year, and the term for the quantile mapping of the estimated and observed data is from 2008 to 2018, excluding the estimation year. In the application to the climate model outputs, the term for training and quantile mapping is from 2008 to 2018. The d4PDF of the ensemble 1 data (over 60 years) was estimated by the training and quantile mapping.

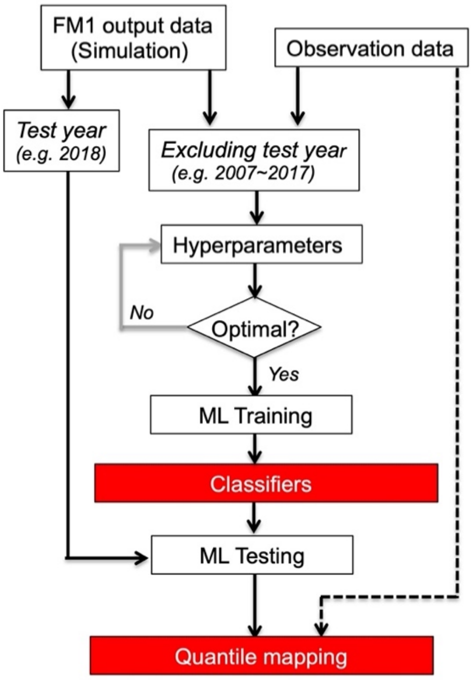

Fig. S2: **Workflow of the downscaling method.**

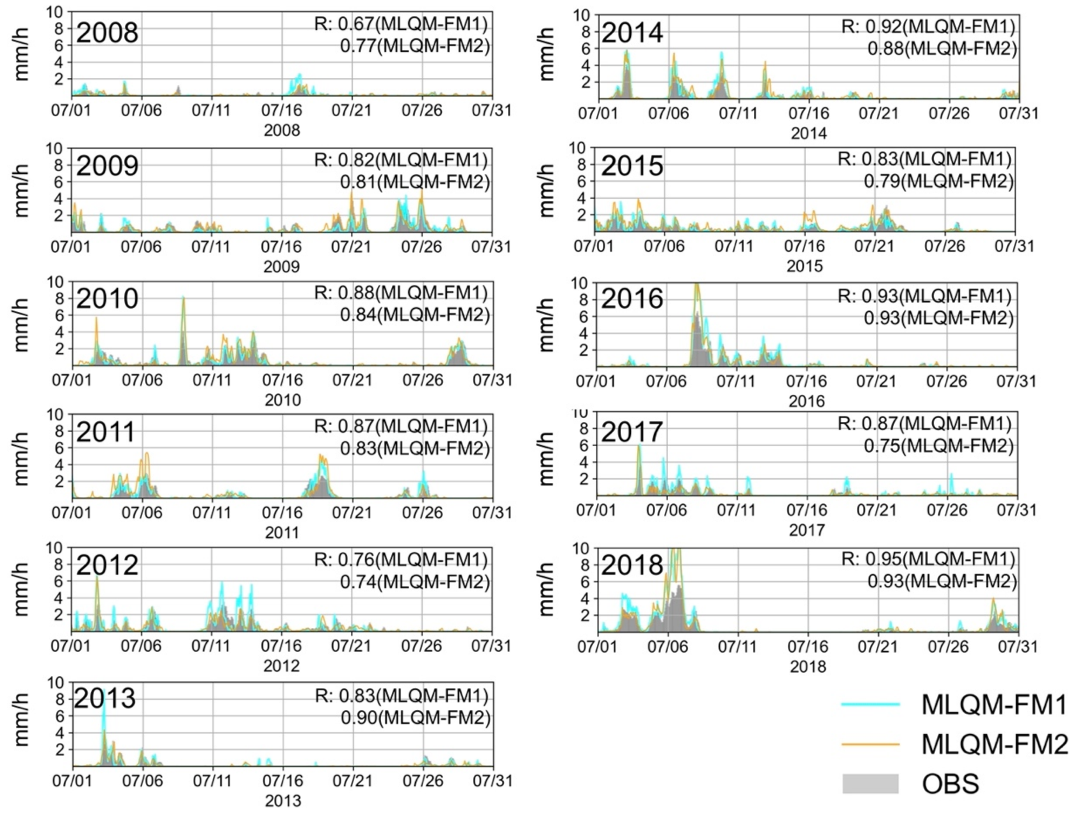

Fig. S3: **Temporal variation in the area-averaged hourly precipitation based on MLQM-FM1, MLQM-FM2, and OBS for July of 2008 to 2018.** The bars represent the observations. The blue and orange lines represent the estimated precipitation using MSM-GPV (MLQM-FM1) and GSM-GPV (MLQM-FM2) as the testing data, respectively. The values in the upper right of each graph indicate the correlation coefficient with the observed values

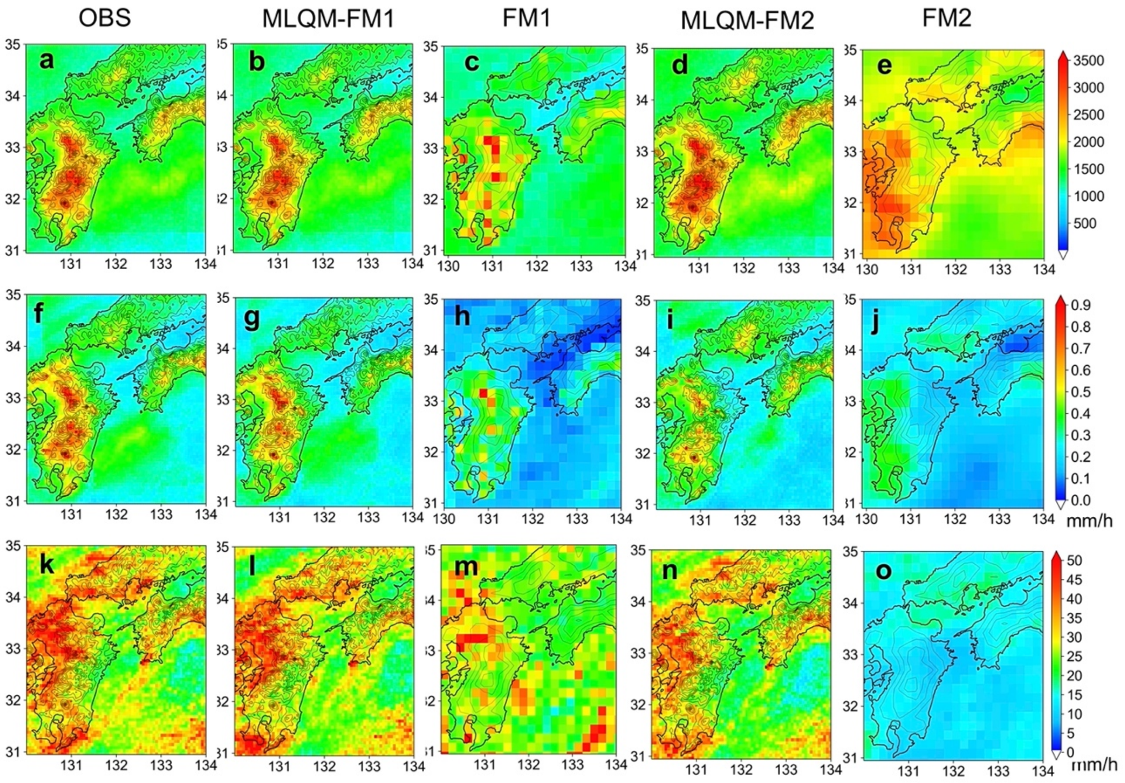

Fig. S4: **Validation of the spatial distribution of estimated precipitation.** Distributions of precipitation frequency greater than or equal to 1 mm/h for the **a,** observation (OBS), **b,** precipitation estimated using the MSM-GPV (MLQM-FM1), **c,** precipitation simulated using the MSM-GPV (FM1), **d,** precipitation estimated using the GSM-GPV (MLQM-FM2), and **e,** precipitation simulated using the GSM-GPV (FM2). **f, g, h, i, and j,** Distributions of monthly precipitation. **k, l, m, n, and o,** Distributions of the 99th percentile values of hourly precipitation. The figures were created using Python 3.6. The maps were created using python3-matplotlib (version 3.7.1, https://matplotlib.org/) and cartopy (version 0.21.1,

https://scitools.org.uk/cartopy). Topographic data of U.S. Geological Survey (USGS) (http://www.usgs.gov) and Japan Meteorological Agency (JMA) were used. Made with Natural Earth. Free vector and raster map data @ naturalearthdata.com. (http://www.naturalearthdata.com/about/terms-of-use/).

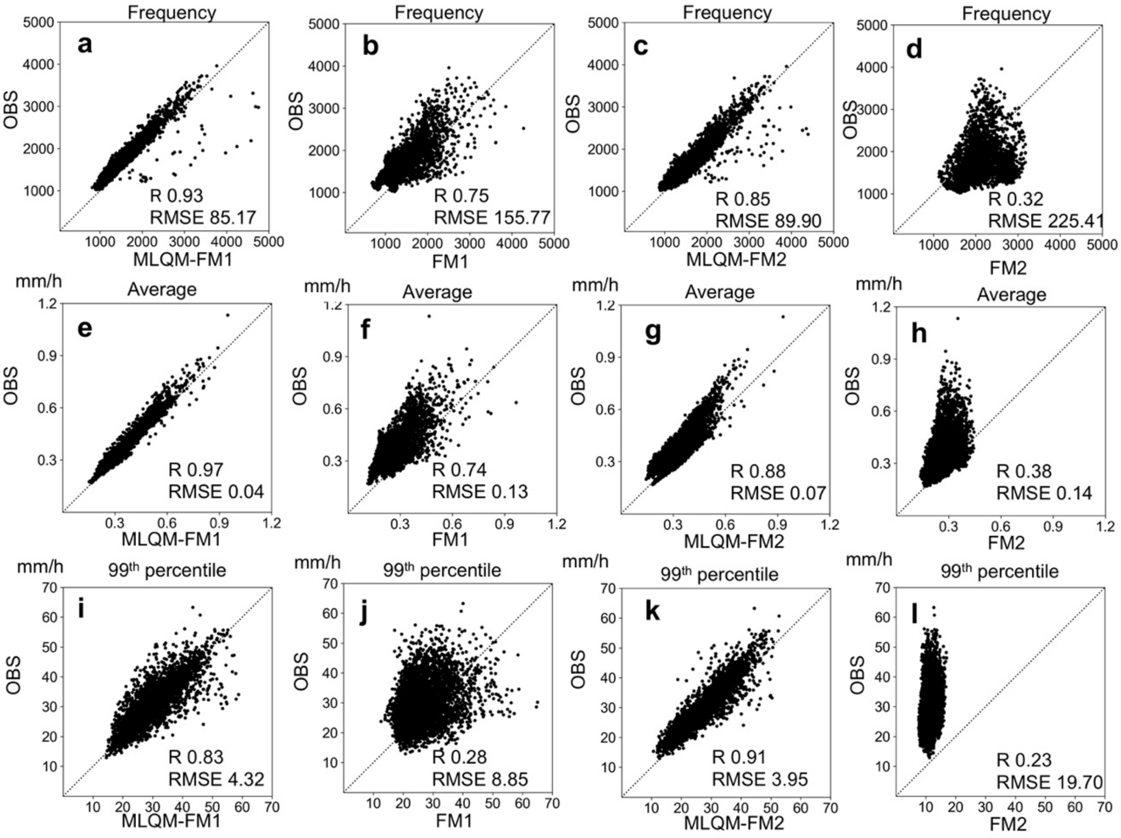

Fig. S5: **Validation of the machine learning-based downscaling method using weather forecast models compared with observations.** Relationship of the hourly precipitation frequency greater than or equal to 1 mm/h for **a,** precipitation estimated by the MSM-GPV (MLQM-FM1), **b,** precipitation simulated by the MSM-GPV (FM1), **c,** precipitation estimated by the GSM-GPV (MLQM-FM2), and **d,** precipitation simulated by the GSM-GPV (FM2) with the OBS data. **e, f, g, and h,** Monthly precipitation. **i, j, k, and l,** 9^{9t}h percentile values of hourly precipitation. R, correlation coefficient; RMSE, root mean square error. The figures were created using Python 3.6.

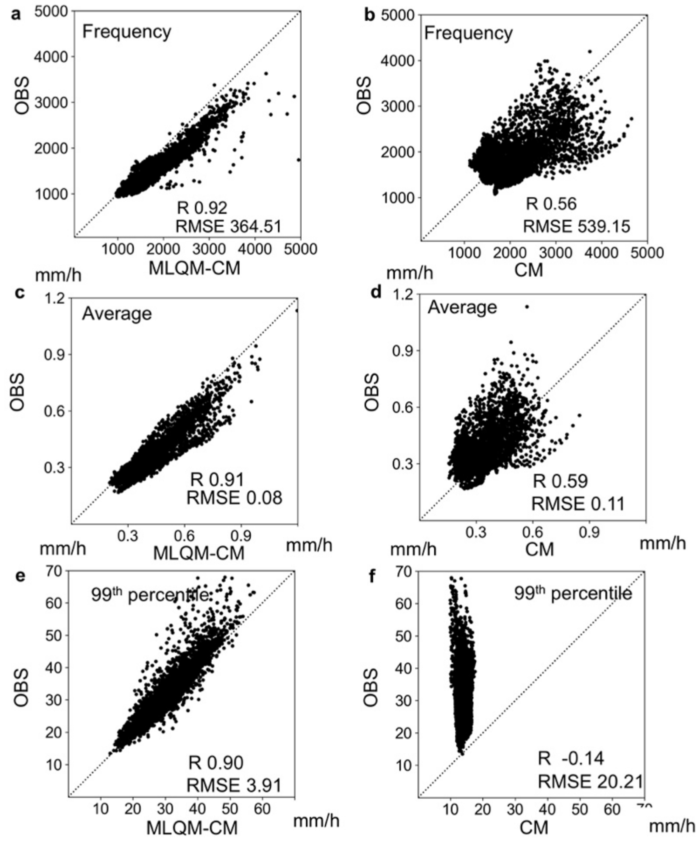

Fig. S6: **Validation of estimated precipitation.** Relationship of the frequency between observations and **a,** MLQM-CM and **b,** CM. **c and d,** Relationship with monthly precipitation. **e and f,** Relationship with the 99th percentile values of hourly precipitation for 1982 to 2011. The OBS data are from 2007 to 2018, and the frequency was adjusted by extending the term 2.5 times.

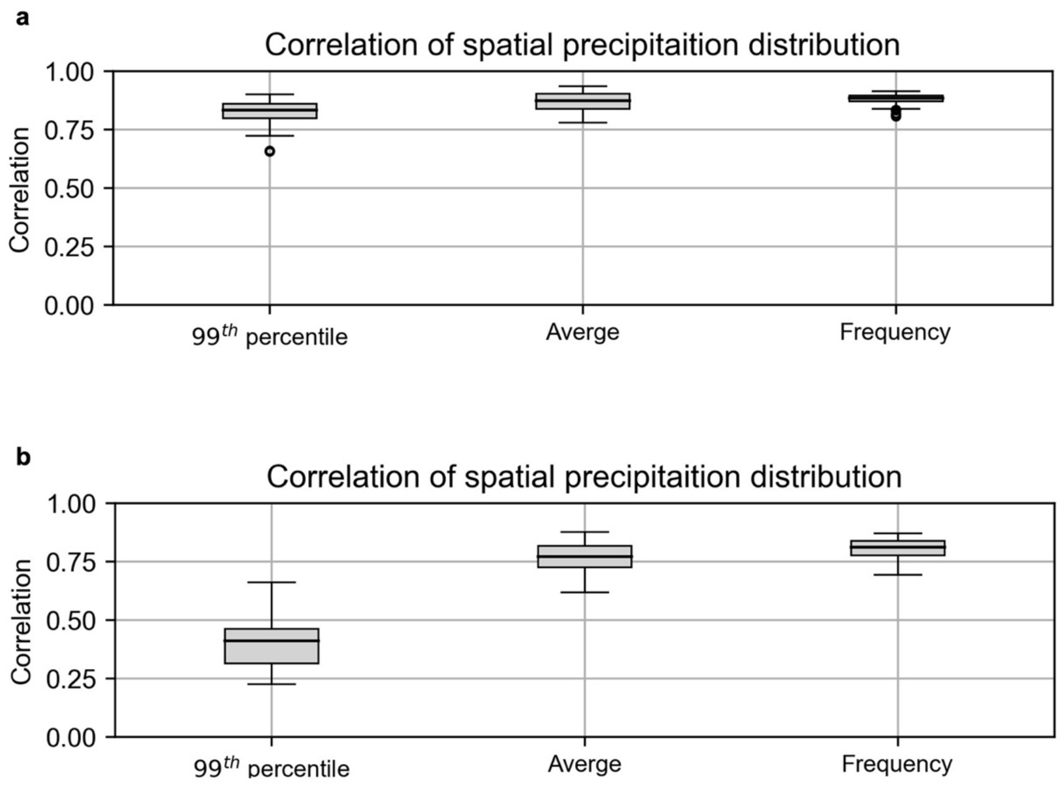

Fig. S7: **Correlation of spatial distribution of 99th percentile, monthly average, and frequency of precipitation between observations and 50 ensemble runs in MLQM-CM. a,** Correlations of the MLQM-CM with OBS. **b,** Correlations of the MLQM-CM with OBS-station.

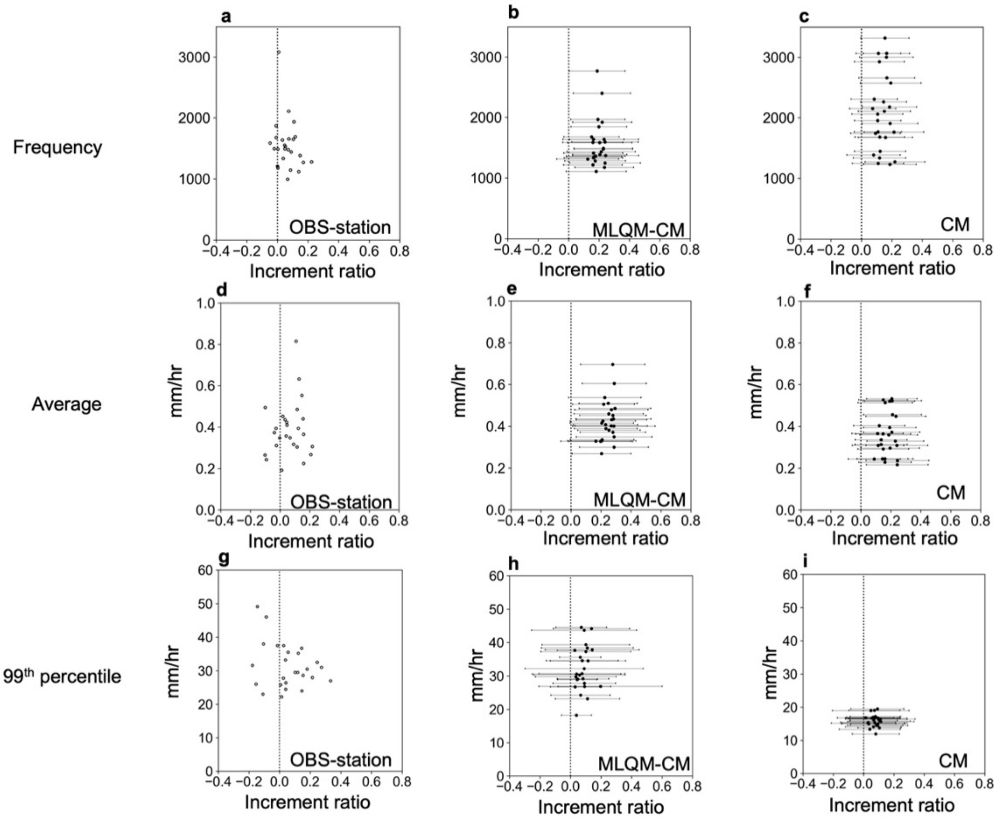

Fig. S8: **Increment ratios of precipitation for 1952–1981 to 1982–2011.** Increment ratios of the frequency, monthly average, and 99th percentile values of hourly precipitation in **a, d, and g**, OBS-station, **b, e, h,** MLQM-CM, and, **c, f, and i,** CM. The black circle markers indicate the ensemble average. The error bars are the standard deviation of the increment ratio for 50 ensembles.

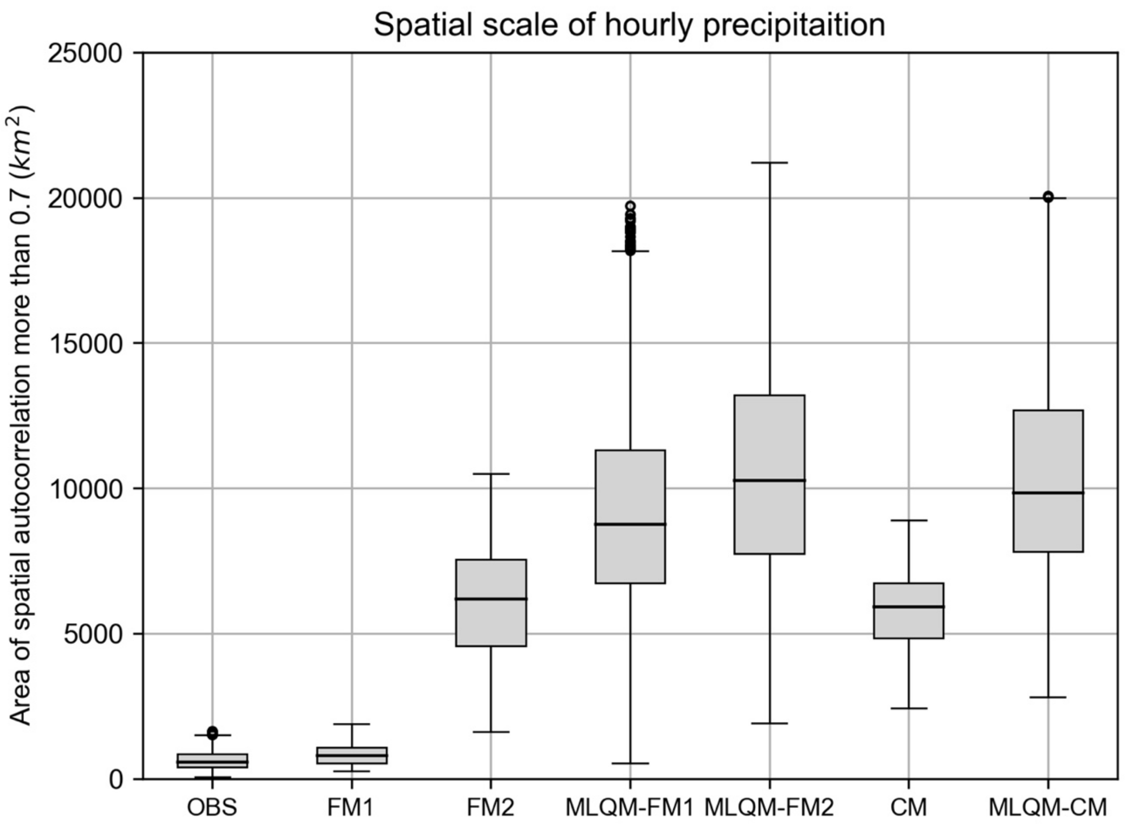

Fig. S9: **Averaged spatial scale of hourly precipitation.** The number of grid points (0.06°-squared area) with averaged spatiotemporal correlations >0.7.

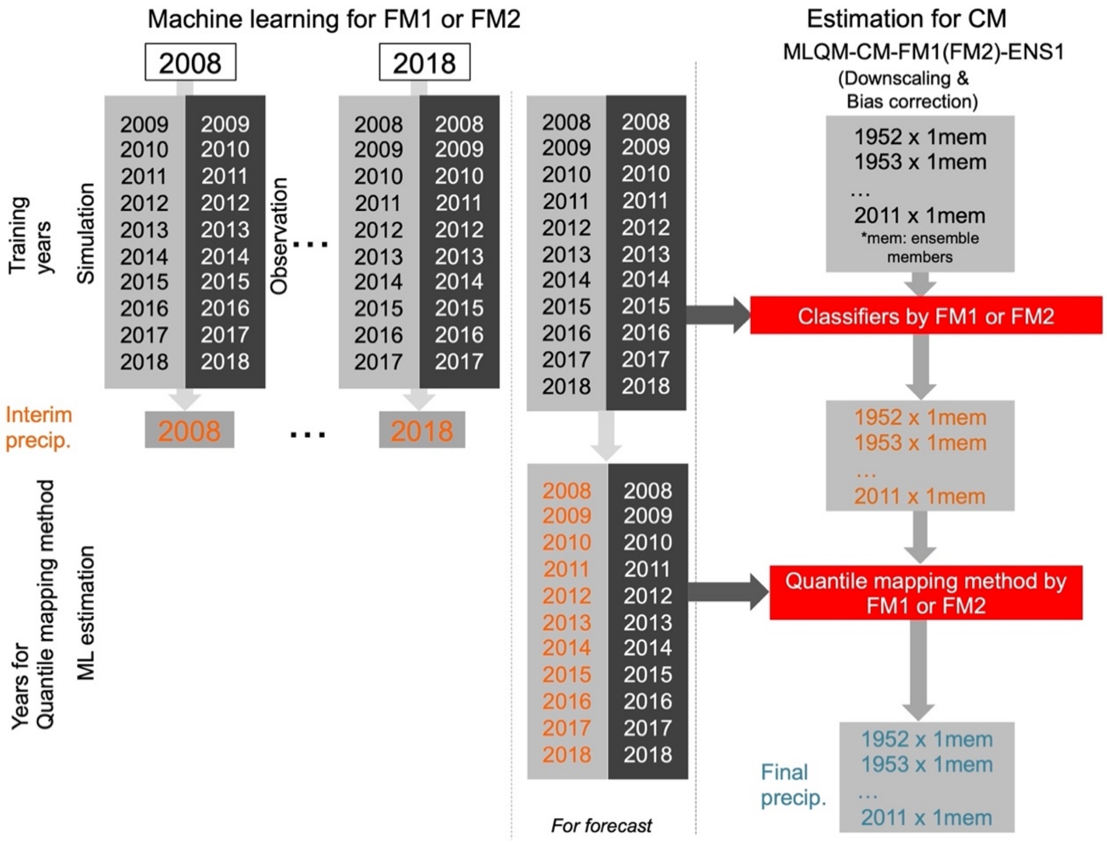

Fig. S10: **Procedure for estimating precipitation using the machine learning-based downscaling method for validation of upscaled data in FM1.**

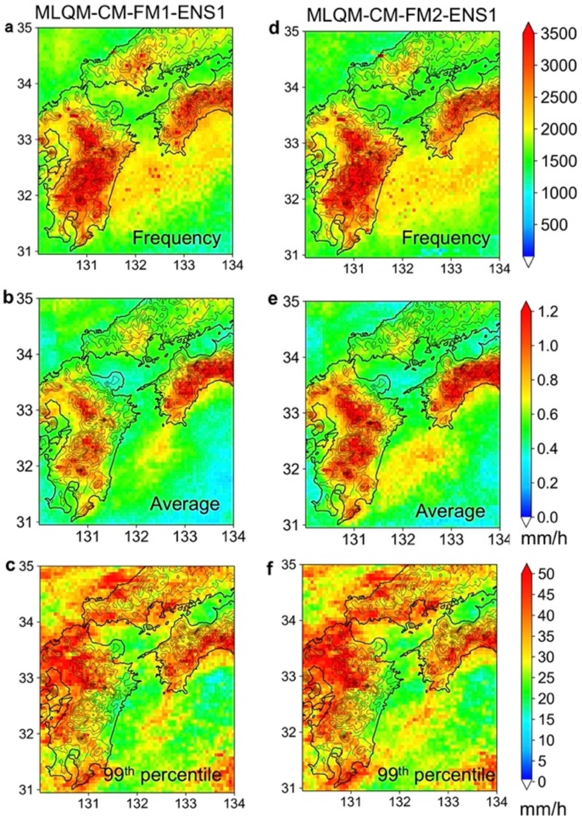

Fig. S11: **Validation of estimated precipitation using upscaled data. a and d,** Frequency of precipitation greater than or equal to 1 mm h^{-1}, **b and e,** monthly mean, and **c and f,** 99th percentile values from 1982 to 2011 for MLQM-CM-FM1-ENS1 (left figures) and MLQM-CM-FM1-ENS2 (right figures). The maps were created using python3-matplotlib (version 3.7.1, https://matplotlib.org/) and cartopy (version 0.21.1, https://scitools.org.uk/cartopy). Topographic data of U.S. Geological Survey (USGS) (http://www.usgs.gov) and Japan Meteorological Agency (JMA) were used. Made with Natural Earth. Free vector and raster map data @ naturalearthdata.com. (http://www.naturalearthdata.com/about/terms-of-use/).

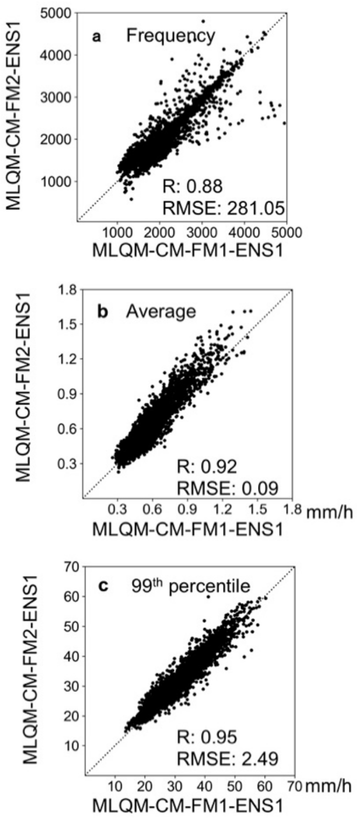

Fig. S12: **Comparison between precipitation estimated using upscaled data and original-resolution data. a,** Precipitation with a frequency greater than or equal to 1 mm h^{-1}, **b,** monthly mean, and **c,** 99th percentile values from 1982 to 2011 for MLQM-CM-FM1-ENS1 and MLQM-CM-FM1-ENS2. R, correlation coefficient; RMSE, root mean square error.

Run types	Training data	Testing data	Training term	Testing term	Term & data for QM
MLQM-FM1*	MSM-GPV (5km → 0.18deg : upscaling)	MSM-GPV (5km → 0.18deg : upscaling)	2007 to 2018	2007 to 2018 (ML estimation1)	2007 to 2018 (ML estimation1 and OBS)
MLQM-FM2	MSM-GPV (5km → 0.18deg : upscaling)	GSM-GPV (20km → 0.18deg)	2007 to 2018	2008 to 2018	2007 to 2018 (ML estimation1 and OBS)
MLQM-CM	MSM-GPV (5km → 0.18deg : upscaling)	d4PDF_RCM (20km → 0.18deg)	2007 to 2018	1952 to 2011 (60 years × 50 ensembles)	2007 to 2018 (ML estimation1 and OBS)
MLQM-CM-FM1-ENS1	MSM-GPV (5km → 0.18deg : upscaling)	d4PDF_RCM (20km → 0.18deg)	2008 to 2018	1952 to 2011 (Ensemble 1) (ML estimation2)	2008 to 2018 (ML estimation2 and OBS)
MLQM-CM-FM2-ENS1	GSM-GPV (20km → 0.18deg)	d4PDF_RCM (20km → 0.18deg)	2008 to 2018	1952 to 2011 (Ensemble 1) (ML estimation3)	2008 to 2018 (ML estimation3 and OBS)

* The estimated data from 2008 to 2018 was used for the analysis

Table S1: **Details of each experiment.**
